# Urachus Pervious after Birth

**Published:** 1866-10

**Authors:** 


### Urachus Pervious after Birth.

Dr. G. J. Townsend relates (*Boston Medical and Surgical Journal*, Sept. 6, 1866,) the following case of this: "I was asked to see a little negro five days old, of mixed parentage, and was told he was passing his water through his belly. On inspection, sure enough, every time the infant cried or made any great exertion, the urine bubbled freely from the umbilicus. The cord had separated normally, and the child was in every other respect vigorous and healthy. There was very evident ulceration of the surface, left by the separation of the cord. The question whether the urethra was pervious was solved at the time of the visit in the affirmative, a fair stream spirting forth *per vias naturales.*

The presence of ulceration at the orifice of the abnormal duct rendered the process of obliterating it very simple. The ulcerated surface was freely cauterized, and the edges of the opening were brought into close apposition and kept there by a strip of adhesive plaster, firmly applied in a longitudinal direction. This was still further secured by a compress of cork covered with wash leather, and kept in place by being stitched to a close-fitting swathe.

The presence of ulceration in this case may be thought to have some bearing upon the question as to the manner in which the cord separates, whether by a process of ulceration or absorption. But the ulceration was evidently an accident here, and caused by the acrid fluid passing constantly over a new and delicate surface, and was healed at once by the arrest of the flow. The patient was well in four days, when the swathe was removed.

Cases of this kind are believed to be very rare, the urachus shriveling up in the human fœtus in the earlier stages of fœtal life."

